# Reassortant Avian Influenza A(H9N2) Viruses in Chickens in Retail Poultry Shops, Pakistan, 2009–2010

**DOI:** 10.3201/eid2104.141570

**Published:** 2015-04

**Authors:** Mamoona Chaudhry, Angélique Angot, Hamad B. Rashid, Giovanni Cattoli, Manzoor Hussain, Giulia Trovò, Alessandra Drago, Viviana Valastro, Michael Thrusfield, Sue Welburn, Mark C. Eisler, Ilaria Capua

**Affiliations:** University of Veterinary and Animal Sciences, Lahore, Pakistan (M. Chaudhry, H.B. Rashid);; University of Edinburgh, Edinburgh, Scotland, UK (M. Chaudhry, M. Thrusfield, S. Welburn);; Istituto Zooprofilattico Sperimentale delle Venezie, Viale dell'Università, Legnaro, Padua, Italy (A. Angot, G. Cattoli, G. Trovò, A. Drago, V. Valastro, I. Capua);; Food and Agriculture Organization of the United Nations, Islamabad, Pakistan (M. Hussain); University of Bristol, Bristol, UK (M.C. Eisler)

**Keywords:** Influenza A virus, avian influenza, reassortant, H9N2 subtype, live bird retail shops, poultry, phylogenetic analysis, zoonoses, viruses, Pakistan, influenza

## Abstract

Phylogenetic analysis of influenza viruses collected during December 2009–February 2010 from chickens in live poultry retail shops in Lahore, Pakistan, showed influenza A(H9N2) lineage polymerase and nonstructural genes generate through inter- and intrasubtypic reassortments. Many amino acid signatures observed were characteristic of human isolates; hence, their circulation could enhance inter- or intrasubtypic reassortment.

The first outbreak of illness caused by avian influenza A(H9N2) virus in Pakistan was reported in 1998; isolates showed a close relationship to subtype H9N2 avian influenza viruses (AIVs) circulating in Hong Kong, China during 1997 that were grouped within the G1 lineage (*1*). In recent years, H9N2 genes have reassorted extensively, generating novel genotypes on the Indian subcontinent. Widespread co-circulation of H9N2 with other AIVs (e.g., highly pathogenic AIVs H5N1 and H7N3) could instigate the generation of novel variant and reassorted viruses, possibly with increased zoonotic potential (*2*).

No information was available about the genetic makeup of AIVs circulating in live poultry retail shops (LPRSs) in Pakistan. This study was conducted to genetically characterize AIVs in LPRSs in Lahore District, Pakistan.

## The Study

We conducted a cross-sectional survey of LPRSs in Lahore (Figure), which is the capital of the Punjab Province in Pakistan. In each of 280 LPRSs, we collected tracheal swab samples from 5 randomly selected chickens and pooled them into 1 composite sample, totaling 280 pooled samples. The samples were characterized at the World Organisation for Animal Health/Food and Agriculture Organization of the United Nations National Reference Laboratory for Avian Influenza in Padua, Italy.

**Figure:**
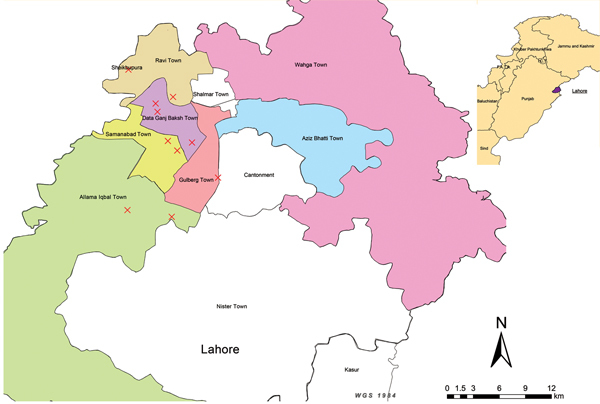
Location of live poultry retail shops (X) in 5 towns in Lahore, Pakistan, where avian influenza A(H9N2) virus isolates were identified in chickens, 2009–2010. Inset shows location of Lahore in Punjab Province.

Of the 280 samples, 10 tested positive for H9N2 subtype by real-time reverse transcription PCR (rRT-PCR) (*3*) and virus isolation test (United Nations www.oie.int/fileadmin/Home/eng/Health_standards/tahm/2.03.04_AI.pdf) (Table 1). Results were negative for H5 and H7 subtypes. Two of the 280 samples were positive for Newcastle disease virus. Each of the 10 H9N2 isolates was characterized by sequencing for 6 gene segments (hemagglutinin [HA]: neuraminidase [NA], nonstructural [NS], matrix [M], and polymerase basic 1 and 2 [PB1, PB2]); 2 isolates (A/chicken/Pakistan/10RS3039-283-11/2010 and A/chicken/Pakistan/10RS3039-289-186/2010) were sequenced for all 8 gene segments (HA, NA, NS, M, PB1, PB2, polymerase [PA], and nucleoprotein [NP]), as described (*4*). Sequences for these viruses were deposited into GenBank (accession nos. KF975457–KF975503, and KP223678–KP223693). The NS gene sequence of A/chicken/Pakistan/10RS3039-284-11/2010 could not be deposited due to poor data quality.

**Table 1:** Influenza A(H9N2) viruses isolated from oropharyngeal swab samples from chickens in live poultry retail shops of Lahore, Pakistan, 2010

Isolates	Collection date	Towns of Lahore District*
A/chicken/Pakistan/10RS3039-283-11/2010	Jan 23	Gulberg Town
A/chicken/Pakistan/10RS3039-284-48/2010	Jan 25	Data Gunj Bakhsh Town
A/chicken/Pakistan/10RS3039-285-63/2010	Jan 26	Data Gunj Bakhsh Town
A/chicken/Pakistan/10RS3039-286-65/2010	Jan 26	Data Gunj Bakhsh Town
A/chicken/Pakistan/10RS3039-287-98/2010	Jan 27	Ravi Town
A/chicken/Pakistan/10RS3039-288-102/2010	Jan 27	Ravi Town
A/chicken/Pakistan/10RS3039-289-186/2010	Feb 11	Samanabad Town
A/chicken/Pakistan/10RS3039-199-199/2010	Feb 11	Samanabad Town
A/chicken/Pakistan/10RS3039-290-230/2010	Feb 13	Allama Iqbal Town
A/chicken/Pakistan/10RS3039-291-266/2010	Feb 14	Allama Iqbal Town
*Lahore District contains the 9 towns and 1 cantonment area.		

We generated neighbor-joining phylogenetic trees for all gene segments using the distance-based method in MEGA version 5.2.2 (http://www.megasoftware.net/). We calculated bootstrap values based on 1,000 replicates of alignment (*5*). HA and NA genes of viruses in this study tightly clustered within the G1 lineage along with H9N2 viruses from Pakistan, India, Iran, Israel, Saudi Arabia, and Bangladesh, suggesting derivation from a common ancestor: A/quail/Hong Kong/G1/97 (Technical Appendix Figure panels A, B). M and NP genes clustered within the G1 lineage (Technical Appendix Figure panels C, D) along with an influenza (H7N3) isolate from Pakistan (A/chicken/Karachi/NARC-100/2004; nucleotide identity >96% for M gene and 95% for NP gene). Two polymerase complex genes, PB1 and PA, and the NS gene did not cluster within G1 lineage or any other established Eurasian lineages: these gene sequences made a separate well-supported cluster with highly similar H9N2 viruses that circulated in Pakistan (2005–2008), Iran, India, and Bangladesh; these sequences had a high sequence identity (>95%) with A/chicken/Karachi/NARC-100/04 (H7N3) (*2*,*6*,*7*) (Technical Appendix Figure panels E, F, G). The PB2 segment clustered separately with H9N2 viruses from the subcontinent and Middle East (Technical Appendix Figure panel H), except A/chicken/Karachi/NARC-100/04. These results indicate a separate Indian subcontinental lineage of H9N2 viruses has emerged (*2*).

On the basis of these analyses, we could conclude that internal genes PB1, PA, and NS of these viruses originated by intersubtypic (between different HA subtypes) reassortment events from local H7N3 viruses circulating in Pakistan (nucleotide identity >95%). This suggests that intersubtypic reassortment events continuously result from mixing of AIV subtypes in domestic poultry and wild birds in Pakistan (*8*), and that PB2, M, and NP genes were acquired by intrasubtypic reassortment between H9N2 viruses of G1 lineage circulating within Pakistan. These results support speculation that the currently circulating H9N2 lineage is a reassortment of G1 lineage from Hong Kong and the highly pathogenic H7N3 virus that circulated in Pakistan and can be assigned to genetic group B (*8*).

We aligned amino acid sequences of current viruses and compared them to representative H9N2 lineages. When compared to the prototype G1 viruses, H9N2 viruses isolated from LPRSs showed that they have evolved to acquire mammalian host-specific mutations throughout the genome (Technical Appendix Table 1). Of these mutations, certain amino acid substitutions throughout the viral genome have become fixed (*9*). All LPRS isolates possessed the K-S-S-R motif at the cleavage site. The presence of lysine at position 4 was observed in H9 isolates from the Indian subcontinent (*2*,*9*), but it is uncommon elsewhere. Amino acid signature changes of human influenza viruses were also observed in internal gene segments (Table 2).

**Table 2:** Amino acid signature changes observed in M1, NP, PB1 and NS1 proteins of influenza A(H9N2) viruses isolated from chickens in live poultry retail shops, Lahore, Pakistan, 2009–2010*

Protein	Amino acid position	Predicted aa		Viruses with detected mutation	Mutation
		Avian	Human		
M1	15	V	I	All 10 isolates analyzed in this study	V15I
NP	372	E	D	Only 2 viruses were analyzed (A/chicken/ Pakistan/10RS3039-283-11/2010 A/chicken/ Pakistan/10RS3039-289-186/2010)	E372D
PB1	13	L	P	All 10 isolates analyzed in this study	L13P
NS1	149	V	A	All 10 isolates analyzed in this study	V149A
	227	E	R or K (H1N1 1918)		E227K
*M, matrix; NP, nucleoprotein; PB, basic polymerase; NS, nonstructured.					

Each of the 10 isolates had the Q^226^L substitution (H3 numbering) in the receptor-binding site of HA, correlating to a shift in affinity from avian-type sialic acid receptor to human-type (*10*). We identified 3 representative substitutions: E/T^190^ A in 9 viruses and E/T^190^V in 1 virus, and Q^227^I in all 10 viruses (H3 numbering). The outcomes of these substitutions have not been investigated; further study is needed (*2*). Glycosylation sites at positions 551, 218, and 206 were absent in the study viruses, suggesting a frequent alteration in sequences from this region (*2*,*6*,*9*) and possibly signifying the selected adaptation of H9N2 to poultry (*10*). We did not find an R^292^K substitution, which is associated with resistance to the sialidase inhibitors oseltamivir and zanamivir, in the NA proteins of any of the 10 LPRS virus isolates. HB sites were also well conserved with few substitutions (K^367^E in 5 viruses, K^367^G in 1 virus, and S^372^A and W^403^R in all 10 viruses). An additional glycosylation site was present at position 44, which is believed to enhance virulence caused by altered antigenicity or sialidase activity (*11*) (Technical Appendix Table 1).

Many residues in nucleoprotein and polymerase are considered determinants of the host range of AIVs and increase virulence or replication in the mammalian host (*2*,*10*,*12*). The analyses of internal genes showed that these viruses also contained mammalian host-specific markers (*2*,*8*) that have become permanent in M protein (M1, V^15^I, T^37^A; M2, E^16^G, L^55^F); in PB1 protein (L^13^P); and in NS1 protein (V^149^A) of all LPRS isolates and in NP (E^372^D) of the 2 isolates we sequenced (Technical Appendix Table 2). In M2 protein, no substitutions linked to resistant amantadine were seen. All viruses contained an uncommon K-S-E-I sequence as a PDZ ligand motif in the NS protein. Residue isoleucine at the C-terminus of PDZ ligand motif has been reported as a rare substitution (*12*). NS also harbored E^227^K mutation in the C-terminal, which has been demonstrated to modulate pathogenicity of AIVs (*13*) and appeared to be a rare amino acid signature, although in this study it was observed in all 10 virus isolates from chickens in LPRSs.

## Conclusions

Our analysis confirmed that continuous gene reassortment has occurred among influenza A(H9N2) viruses since their emergence in poultry in Pakistan. Because H9N2 viruses infect multiple species, they may donate genes to emerging human pathogens; it has been observed that H7N9 acquired internal genes from the avian H9N2 virus (*14*). In wet markets, availability of freshly slaughtered poultry, live poultry transportation, and mixed trading of domestic animals provide a favorable environment for gene reassortment, mutation, and interspecies transmission of AIVs (*15*). Continuous circulation of these viruses in LPRSs increases the chances of their evolution into new genotypes. Close contact of humans and poultry in LPRSs with no biosecurity barriers increases the risk for emergence of novel influenza viruses with zoonotic or human pandemic potential. Continued surveillance in LPRSs is essential to better understand the public health risk posed by H9N2 AIVs.

**Technical Appendix.** Comparisons of structure and pathogenicity determinants of avian influenza isolates and phylogenetic distribution of 10 sequenced viruses from samples from chickens in live poultry retail shops in Lahore, Pakistan, 2009–2010.
