# Supplementary material for: Reassortant Avian Influenza A(H9N2) Viruses in Chickens in Retail Poultry Shops, Pakistan, 2009–2010
Source: Emerg Infect Dis. 2015 Apr;21(4):673–6. doi: 10.3201/eid2104.141570 (PMC4378488; doi:10.3201/eid2104.141570)
Supplement: Supplementary file 1 — Technical Appendix. Comparisons of structure and pathogenicity determinants of avian influenza isolates and phylogenetic distribution of 10 sequenced viruses from samples from chickens in live poultry retail shops in Lahore, Pakistan, 2009–2010. [file 14-1570-Techapp-s1.pdf]

Article DOI: http://dx.doi.org/10.3201/eid2104.141570

Reassortant Avian Influenza A(H9N2) Viruses in Chickens in Retail Poultry Shops, Pakistan, 2009–2010

Technical Appendix

Technical Appendix Table 1. Comparison of critical amino acid substitutions in hemagglutinin and neuraminidase proteins in H9N2 isolates from samples collected from chickens in live poultry retail shops, Pakistan, 2009–2010*

	HA						NA						
	HA RBS					HA1/HA2 cleavage site	Glycosylation site	Stalk deletion				Semiabsorbing site	
Residues at HA RBS (H9N2 numbering)	191	198	234	235	236	335-338	218	38-39	46-50	62-64	372	402	403
H3 Residues at HA RBS (H3 numbering)	183	190	226	227	228	326-329	210	–	–	–	–	–	–
A/Qa/HK/G1/97	H	E	L	Q	G	RSSR	Yes	Yes	No	No	S	I	R
A/duck/HK/Y280/97[2]	N	T	L	Q	G	RSSR	Yes	No	No	No	S	I	R
A/ck/Korea/38349-p96323/96[3]	H	E	Q	Q	G	ASYR	Yes	No	No	No	S	N	W
A/Hong Kong/1073/99[4]	H	E	L	Q	G	RSSR	Yes	Yes	No	No	S	N	W
A/ck/Pak/UDL-01/07[5]	H	A	L	I	G	RSSR	No	No	No	No	S	N	W
A/ck/Pak/10RS3039-283-11/2010	H	V	L	I	G	KSSR	No	No	No	No	A	N	R
A/ck/ Pak/10RS3039-284-48/2010	H	A	L	I	G	KSSR	No	No	No	No	A	N	R
A/ck/Pak/10RS3039-285-63/2010	H	A	L	I	G	KSSR	No	No	No	No	A	N	R
A/ck/ Pak/10RS3039-286-65/2010	H	A	L	I	G	KSSR	No	No	No	No	A	N	R
A/ck/ Pak/10RS3039-287-98/2010	H	A	L	I	G	KSSR	No	No	No	No	A	N	R
A/ck/ Pak/10RS3039-288-102/2010	H	A	L	I	G	KSSR	No	No	No	No	A	N	R
A/ck/ Pak/10RS3039-289-186/2010	H	A	L	I	G	KSSR	No	No	No	No	A	N	R
A/ck/ Pak/10RS3039-199-199/2010	H	A	L	I	G	KSSR	No	No	No	No	A	N	R
A/ck/ Pak/10RS3039-290-230/2010	H	A	L	I	G	KSSR	No	No	No	No	A	N	R
A/ck/ Pak/10RS3039-291-266/2010	H	A	L	I	G	KSSR	No	No	No	No	A	N	R

*Residual differences and similarities are compared with reference strains of lineages: G1-lineage (1), Y280-like (2), and Korea-like (3), vaccine strain (4), and previous H9N2 isolate originating in Pakistan (5).HA, hemagglutinin,; NA, neuraminidase; RBS, receptor binding site; –, not applicable; A, alanine; D, aspartic acid; E, glutamic acid; G, glycine; H, histidine; I, isoleucine; K, lysine; L, leucine; N, asparagine; Q, glutamine; R, arginine; S, serine; T, threonine; V, valine.

Technical Appendix Table 2. Comparison with validated host-specific amino acids residue and pathogenicity determinants in the M1, NP, PB2, PB1, and NS1 protein in H9N2 viruses isolated from 280 composite samples in 280 live poultry retail shops of Lahore, Pakistan*

	M1		M2			M2 drug resistance			NS		NP					PA		PA-X		PB1		PB1-F2					PB2	
x	15	16	20	28	55	26	31	Deletion of amino acid 80-84	NS1 (227)	PL motif	33	109	214	313	372	57	409	108	134	13	336	Size (aa)	73	79	82	87	256	355
Predicted Avian	V	E	S	I	L	NA	NA	NA	E	NA	V	I	R	F	E	R	T	D	K	L	V		K	R	L	E	D	K
Predicted Mammalian	I	G/D	N	I/V	F	NA	NA	NA	R/K	NA	I	V	K	Y	D	Q	S			P	I		R	Q	S	G	G	Q
Predicted amantadine resistant	NA	NA	NA	NA	NA	F	N/G	NA	NA	NA	NA	NA	NA	NA	NA	NA	NA	NA	NA	NA	NA	NA	NA	NA	NA	NA	NA	NA
Predicted amantadine sensitive	NA	NA	NA	NA	NA	L	S	NA	NA	NA	NA	NA	NA	NA	NA	NA	NA	NA	NA	NA	NA	NA	NA	NA	NA	NA	NA	NA
A/Qa/HK/G1/97[1]	U	U	U	U	U	U	U	No	E	EPEV	U	U	U	U	U	U	U	U	U	U	U	U	U	U	U	U	U	U
A/duck/HK/Y280/97[2]	U	U	U	U	U	U	U		E	EPEV	U	U	U	U	U	U	U	U	U	U	U	U	U	U	U	U	U	U
A/Hong Kong/1073/99[3]	I								E	EPEV		I	R	F	E	R	S			P	V							
A/ck/ Pak/10RS3039-283-11/2010	I	**G**	K	I	**F**	L	S	No	**K**	**K**SEI	V	I	R	F	**D**	R	S	D	K	**P**	V	90	K	R	L	E	D	R
A/ck/ Pak/10RS3039-284-48/2010	I	**G**	K	I	**F**	L	S		**K**	**K**SEI	V	I	R	F	**D**	R	S	D	K	**P**	V	90	K	R	L	E	D	R
A/ck/ Pak/10RS3039-285-63/2010	I	**G**	K	I	**F**	L	S		**K**	**K**SEI	V	I	R	F	**D**	R	S	D	K	**P**	V	90	K	R	L	E	D	R
A/ck/ Pak/10RS3039-286-65/2010	I	**G**	K	I	**F**	L	S		**K**	**K**SEI	V	I	R	F	**D**	R	S	D	K	**P**	V	90	K	R	L	E	D	R
A/ck/ Pak/10RS3039-287-98/2010	I	**G**	K	I	**F**	L	S		**K**	**K**SEI	V	I	R	F	**D**	R	S	D	K	**P**	V	90	K	R	L	E	D	R
A/ck/ Pak/10RS3039-288-102/2010	I	**G**	K	I	**F**	L	S		**K**	**K**SEI	V	I	R	F	**D**	R	S	D	K	**P**	V	90	K	R	L	E	D	R
A/ck/ Pak/10RS3039-289-186/2010	I	**G**	K	I	**F**	L	S		**K**	**K**SEI	V	I	R	F	**D**	R	S	D	K	**P**	V	90	K	R	L	E	D	R
A/ck/ Pak/10RS3039-199-199/2010	I	**G**	K	I	**F**	L	S		**K**	**K**SEI	V	I	R	F	**D**	R	S	D	K	**P**	V	90	K	R	L	E	D	R
A/ck/ Pak/10RS3039-290-230/2010	I	**G**	K	I	**F**	L	S		**K**	**K**SEI	V	I	R	F	**D**	R	S	D	K	**P**	V	90	K	R	L	E	D	R
A/ck/ Pak/10RS3039-291-266/2010	I	**G**	K	I	**F**	L	S		**K**	**K**SEI	V	I	R	F	**D**	R	S	D	K	**P**	V	90	K	R	L	E	D	R

*Residues differences and similarities are compared with reference strains of lineages (G1-lineage[1], Y280-like[2]), and vaccine strain[3]. Amino acids in boldface indicate the host-associated amino acid signature changes identical to those in influenza viruses isolated from humans. A, alanine; D, aspartic acid; E, glutamic acid; G, glycine; H, histidine; I, isoleucine; K, lysine; L, leucine; N, asparagine; Q, glutamine; R, arginine; S, serine; T, threonine; V, valine; NA, Not applicable; ND, No data; U, unknown.

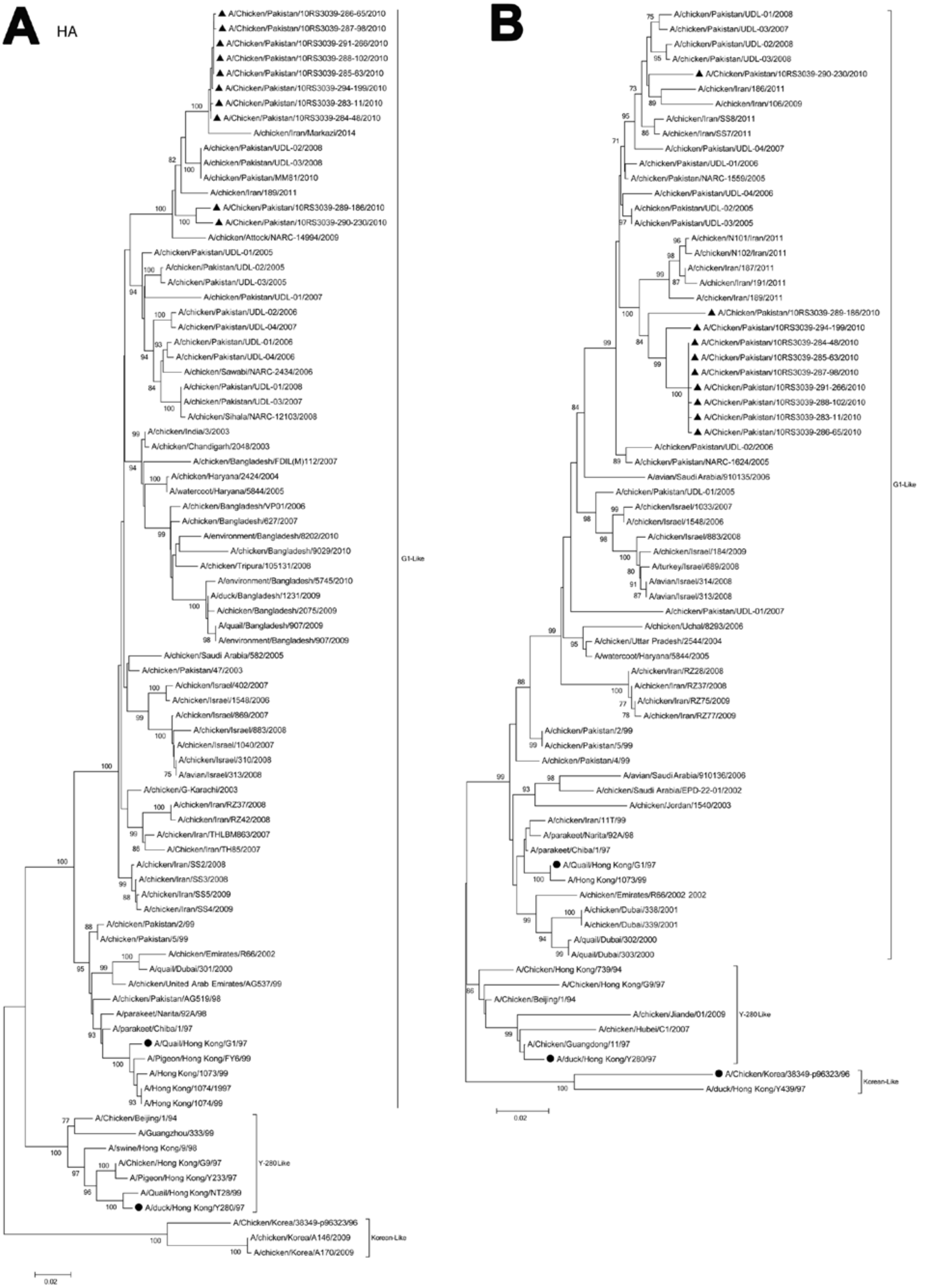

A
HA
A/Chicken/Pakistan/10RS3039-286-65/2010
A/Chicken/Pakistan/10RS3039-287-98/2010
A/Chicken/Pakistan/10RS3039-291-266/2010
A/Chicken/Pakistan/10RS3039-288-102/2010
A/Chicken/Pakistan/10RS3039-285-63/2010
A/Chicken/Pakistan/10RS3039-294-199/2010
A/Chicken/Pakistan/10RS3039-283-11/2010
A/Chicken/Pakistan/10RS3039-284-48/2010
A/chicken/Iran/Markazi/2014
A/chicken/Pakistan/UDL-02/2008
A/chicken/Pakistan/UDL-03/2008
A/chicken/Pakistan/MM81/2010
A/chicken/Iran/189/2011
A/Chicken/Pakistan/10RS3039-289-186/2010
A/Chicken/Pakistan/10RS3039-290-230/2010
A/chicken/Attock/NARC-14994/2009
A/chicken/Pakistan/UDL-01/2005
A/chicken/Pakistan/UDL-02/2005
A/chicken/Pakistan/UDL-03/2005
A/chicken/Pakistan/UDL-01/2007
A/chicken/Pakistan/UDL-02/2006
A/chicken/Pakistan/UDL-04/2007
A/chicken/Pakistan/UDL-01/2006
A/chicken/Pakistan/UDL-04/2006
A/chicken/Sawabi/NARC-2434/2006
A/chicken/Pakistan/UDL-01/2008
A/chicken/Pakistan/UDL-03/2007
A/chicken/Sihala/NARC-12103/2008
A/chicken/India/3/2003
A/chicken/Chandigarh/2048/2003
A/chicken/Bangladesh/FDIL(M)112/2007
A/chicken/Haryana/2424/2004
A/watercoot/Haryana/5844/2005
A/chicken/Bangladesh/VP01/2006
A/chicken/Bangladesh/627/2007
A/environment/Bangladesh/8202/2010
A/chicken/Bangladesh/9029/2010
A/chicken/Tripura/105131/2008
A/environment/Bangladesh/5745/2010
A/duck/Bangladesh/1231/2009
A/chicken/Bangladesh/2075/2009
A/quail/Bangladesh/907/2009
A/environment/Bangladesh/907/2009
A/chicken/Saudi Arabia/582/2005
A/chicken/Pakistan/47/2003
A/chicken/Israel/402/2007
A/chicken/Israel/1548/2006
A/chicken/Israel/869/2007
A/chicken/Israel/883/2008
A/chicken/Israel/1040/2007
A/chicken/Israel/310/2008
A/avian/Israel/313/2008
A/chicken/G-Karachi/2003
A/chicken/Iran/RZ37/2008
A/chicken/Iran/RZ42/2008
A/chicken/Iran/THLBM863/2007
A/Chicken/Iran/TH85/2007
A/chicken/Iran/SS2/2008
A/chicken/Iran/SS3/2008
A/chicken/Iran/SS5/2009
A/chicken/Iran/SS4/2009
A/chicken/Pakistan/2/99
A/chicken/Pakistan/5/99
A/chicken/Emirates/R66/2002
A/quail/Dubai/301/2000
A/chicken/United Arab Emirates/AG537/99
A/chicken/Pakistan/AG519/98
A/parakeet/Narita/92A/98
A/parakeet/Chiba/1/97
A/Quail/Hong Kong/G1/97
A/Pigeon/Hong Kong/FY6/99
A/Hong Kong/1073/99
A/Hong Kong/1074/1997
A/Hong Kong/1074/99
G1-Like
A/Chicken/Beijing/1/94
A/Guangzhou/333/99
A/swine/Hong Kong/9/98
A/Chicken/Hong Kong/G9/97
A/Pigeon/Hong Kong/Y233/97
A/Quail/Hong Kong/NT28/99
A/duck/Hong Kong/Y280/97
Y-280 Like
A/Chicken/Korea/38349-p96323/96
A/chicken/Korea/A146/2009
A/chicken/Korea/A170/2009
Korean-Like
0.02
B
A/chicken/Pakistan/UDL-01/2008
A/chicken/Pakistan/UDL-03/2007
A/chicken/Pakistan/UDL-02/2008
A/chicken/Pakistan/UDL-03/2008
A/Chicken/Pakistan/10RS3039-290-230/2010
A/chicken/Iran/186/2011
A/chicken/Iran/106/2009
A/chicken/Iran/SS8/2011
A/chicken/Iran/SS7/2011
A/chicken/Pakistan/UDL-04/2007
A/chicken/Pakistan/UDL-01/2006
A/chicken/Pakistan/NARC-1559/2005
A/chicken/Pakistan/UDL-04/2006
A/chicken/Pakistan/UDL-02/2005
A/chicken/Pakistan/UDL-03/2005
A/chicken/N101/Iran/2011
A/chicken/N102/Iran/2011
A/chicken/Iran/187/2011
A/chicken/Iran/191/2011
A/chicken/Iran/189/2011
A/Chicken/Pakistan/10RS3039-289-186/2010
A/Chicken/Pakistan/10RS3039-294-199/2010
A/Chicken/Pakistan/10RS3039-284-48/2010
A/Chicken/Pakistan/10RS3039-285-63/2010
A/Chicken/Pakistan/10RS3039-287-98/2010
A/Chicken/Pakistan/10RS3039-291-266/2010
A/Chicken/Pakistan/10RS3039-288-102/2010
A/Chicken/Pakistan/10RS3039-283-11/2010
A/Chicken/Pakistan/10RS3039-286-65/2010
A/chicken/Pakistan/UDL-02/2006
A/chicken/Pakistan/NARC-1624/2005
A/avian/Saudi Arabia/910135/2006
A/chicken/Pakistan/UDL-01/2005
A/chicken/Israel/1033/2007
A/chicken/Israel/1548/2006
A/chicken/Israel/883/2008
A/chicken/Israel/184/2009
A/turkey/Israel/689/2008
A/avian/Israel/314/2008
A/avian/Israel/313/2008
A/chicken/Pakistan/UDL-01/2007
A/chicken/Uchal/8293/2006
A/chicken/Uttar Pradesh/2544/2004
A/watercoot/Haryana/5844/2005
A/chicken/Iran/RZ28/2008
A/chicken/Iran/RZ37/2008
A/chicken/Iran/RZ75/2009
A/chicken/Iran/RZ77/2009
A/chicken/Pakistan/2/99
A/chicken/Pakistan/5/99
A/chicken/Pakistan/4/99
A/avian/Saudi Arabia/910136/2006
A/chicken/Saudi Arabia/EPD-22-01/2002
A/chicken/Jordan/1540/2003
A/chicken/Iran/11T/99
A/parakeet/Narita/92A/98
A/parakeet/Chiba/1/97
A/Quail/Hong Kong/G1/97
A/Hong Kong/1073/99
A/chicken/Emirates/R66/2002 2002
A/chicken/Dubai/338/2001
A/chicken/Dubai/339/2001
A/quail/Dubai/302/2000
A/quail/Dubai/303/2000
G1-Like
A/Chicken/Hong Kong/739/94
A/Chicken/Hong Kong/G9/97
A/Chicken/Beijing/1/94
A/chicken/Jiande/01/2009
A/chicken/Hubei/C1/2007
A/Chicken/Guangdong/11/97
A/duck/Hong Kong/Y280/97
Y-280 Like
A/Chicken/Korea/38349-p96323/96
A/duck/Hong Kong/Y439/97
Korean-Like
0.02

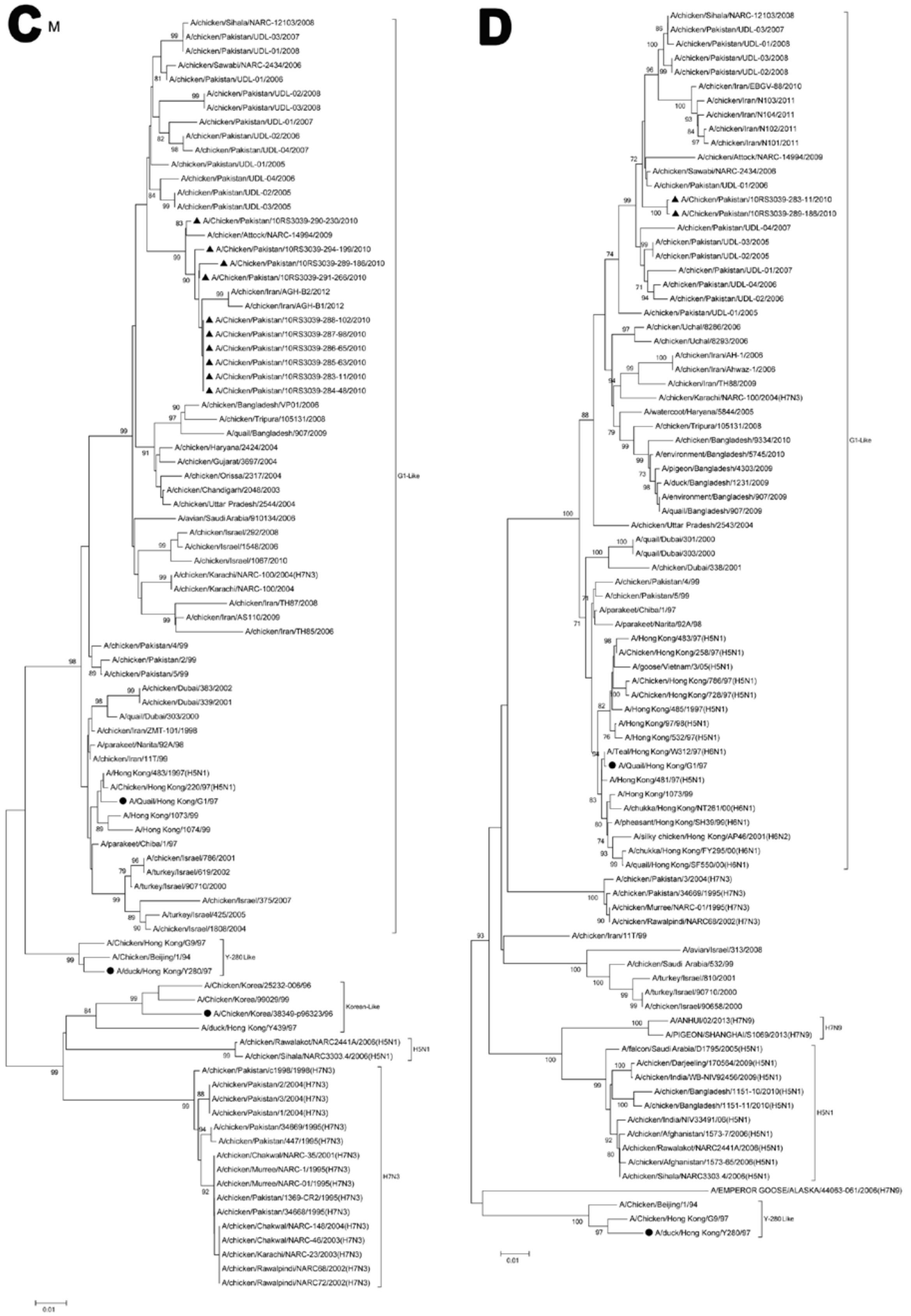
C
M
A/chicken/Sihala/NARC-12103/2008
A/chicken/Pakistan/UDL-03/2007
A/chicken/Pakistan/UDL-01/2008
A/chicken/Sawabi/NARC-2434/2006
A/chicken/Pakistan/UDL-01/2006
A/chicken/Pakistan/UDL-02/2008
A/chicken/Pakistan/UDL-03/2008
A/chicken/Pakistan/UDL-01/2007
A/chicken/Pakistan/UDL-02/2006
A/chicken/Pakistan/UDL-04/2007
A/chicken/Pakistan/UDL-01/2005
A/chicken/Pakistan/UDL-04/2006
A/chicken/Pakistan/UDL-02/2005
A/chicken/Pakistan/UDL-03/2005
A/Chicken/Pakistan/10RS3039-290-230/2010
A/chicken/Attock/NARC-14994/2009
A/Chicken/Pakistan/10RS3039-294-199/2010
A/Chicken/Pakistan/10RS3039-289-186/2010
A/Chicken/Pakistan/10RS3039-291-266/2010
A/chicken/Iran/AGH-B2/2012
A/chicken/Iran/AGH-B1/2012
A/Chicken/Pakistan/10RS3039-288-102/2010
A/Chicken/Pakistan/10RS3039-287-98/2010
A/Chicken/Pakistan/10RS3039-286-65/2010
A/Chicken/Pakistan/10RS3039-285-63/2010
A/Chicken/Pakistan/10RS3039-283-11/2010
A/Chicken/Pakistan/10RS3039-284-48/2010
A/chicken/Bangladesh/VP01/2006
A/chicken/Tripura/105131/2008
A/quail/Bangladesh/907/2009
A/chicken/Haryana/2424/2004
A/chicken/Gujarat/3697/2004
A/chicken/Orissa/2317/2004
A/chicken/Chandigarh/2048/2003
A/chicken/Uttar Pradesh/2544/2004
A/avian/Saudi Arabia/910134/2006
A/chicken/Israel/292/2008
A/chicken/Israel/1548/2006
A/chicken/Israel/1067/2010
A/chicken/Karachi/NARC-100/2004(H7N3)
A/chicken/Karachi/NARC-100/2004
A/chicken/Iran/TH87/2008
A/chicken/Iran/AS110/2009
A/chicken/Iran/TH85/2006
A/chicken/Pakistan/4/99
A/chicken/Pakistan/2/99
A/chicken/Pakistan/5/99
A/chicken/Dubai/383/2002
A/chicken/Dubai/339/2001
A/quail/Dubai/303/2000
A/chicken/Iran/ZMT-101/1998
A/parakeet/Narita/92A/98
A/chicken/Iran/11T/99
A/Hong Kong/483/1997(H5N1)
A/Chicken/Hong Kong/220/97(H5N1)
A/Quail/Hong Kong/G1/97
A/Hong Kong/1073/99
A/Hong Kong/1074/99
A/parakeet/Chiba/1/97
A/chicken/Israel/786/2001
A/turkey/Israel/619/2002
A/turkey/Israel/90710/2000
A/chicken/Israel/375/2007
A/turkey/Israel/425/2005
A/chicken/Israel/1808/2004
G1-Like
A/Chicken/Hong Kong/G9/97
A/Chicken/Beijing/1/94
A/duck/Hong Kong/Y280/97
Y-280 Like
A/Chicken/Korea/25232-006/96
A/Chicken/Korea/99029/99
A/Chicken/Korea/38349-p96323/96
A/duck/Hong Kong/Y439/97
Korean-Like
A/chicken/Rawalakot/NARC2441A/2006(H5N1)
A/chicken/Sihala/NARC3303.4/2006(H5N1)
H5N1
A/chicken/Pakistan/c1998/1998(H7N3)
A/chicken/Pakistan/2/2004(H7N3)
A/chicken/Pakistan/3/2004(H7N3)
A/chicken/Pakistan/1/2004(H7N3)
A/chicken/Pakistan/34669/1995(H7N3)
A/chicken/Pakistan/447/1995(H7N3)
A/chicken/Chakwal/NARC-35/2001(H7N3)
A/chicken/Murree/NARC-1/1995(H7N3)
A/chicken/Murree/NARC-01/1995(H7N3)
A/chicken/Pakistan/1369-CR2/1995(H7N3)
A/chicken/Pakistan/34668/1995(H7N3)
A/chicken/Chakwal/NARC-148/2004(H7N3)
A/chicken/Chakwal/NARC-46/2003(H7N3)
A/chicken/Karachi/NARC-23/2003(H7N3)
A/chicken/Rawalpindi/NARC68/2002(H7N3)
A/chicken/Rawalpindi/NARC72/2002(H7N3)
H7N3
0.01
D
A/chicken/Sihala/NARC-12103/2008
A/chicken/Pakistan/UDL-03/2007
A/chicken/Pakistan/UDL-01/2008
A/chicken/Pakistan/UDL-03/2008
A/chicken/Pakistan/UDL-02/2008
A/chicken/Iran/EBGV-88/2010
A/chicken/Iran/N103/2011
A/chicken/Iran/N104/2011
A/chicken/Iran/N102/2011
A/chicken/Iran/N101/2011
A/chicken/Attock/NARC-14994/2009
A/chicken/Sawabi/NARC-2434/2006
A/chicken/Pakistan/UDL-01/2006
A/Chicken/Pakistan/10RS3039-283-11/2010
A/Chicken/Pakistan/10RS3039-289-186/2010
A/chicken/Pakistan/UDL-04/2007
A/chicken/Pakistan/UDL-03/2005
A/chicken/Pakistan/UDL-02/2005
A/chicken/Pakistan/UDL-01/2007
A/chicken/Pakistan/UDL-04/2006
A/chicken/Pakistan/UDL-02/2006
A/chicken/Pakistan/UDL-01/2005
A/chicken/Uchal/8286/2006
A/chicken/Uchal/8293/2006
A/chicken/Iran/AH-1/2006
A/chicken/Iran/Ahwaz-1/2006
A/chicken/Iran/TH88/2009
A/chicken/Karachi/NARC-100/2004(H7N3)
A/watercoot/Haryana/5844/2005
A/chicken/Tripura/105131/2008
A/chicken/Bangladesh/9334/2010
A/environment/Bangladesh/5745/2010
A/pigeon/Bangladesh/4303/2009
A/duck/Bangladesh/1231/2009
A/environment/Bangladesh/907/2009
A/quail/Bangladesh/907/2009
A/chicken/Uttar Pradesh/2543/2004
A/quail/Dubai/301/2000
A/quail/Dubai/303/2000
A/chicken/Dubai/338/2001
A/chicken/Pakistan/4/99
A/chicken/Pakistan/5/99
A/parakeet/Chiba/1/97
A/parakeet/Narita/92A/98
A/Hong Kong/483/97(H5N1)
A/Chicken/Hong Kong/258/97(H5N1)
A/goose/Vietnam/3/05(H5N1)
A/Chicken/Hong Kong/786/97(H5N1)
A/Chicken/Hong Kong/728/97(H5N1)
A/Hong Kong/485/1997(H5N1)
A/Hong Kong/97/98(H5N1)
A/Hong Kong/532/97(H5N1)
A/Teal/Hong Kong/W312/97(H6N1)
A/Quail/Hong Kong/G1/97
A/Hong Kong/481/97(H5N1)
A/Hong Kong/1073/99
A/chukka/Hong Kong/NT261/00(H6N1)
A/pheasant/Hong Kong/SH39/99(H6N1)
A/silky chicken/Hong Kong/AP46/2001(H6N2)
A/chukka/Hong Kong/FY295/00(H6N1)
A/quail/Hong Kong/SF550/00(H6N1)
G1-Like
A/chicken/Pakistan/3/2004(H7N3)
A/chicken/Pakistan/34668/1995(H7N3)
A/chicken/Murree/NARC-01/1995(H7N3)
A/chicken/Rawalpindi/NARC68/2002(H7N3)
A/chicken/Iran/11T/99
A/avian/Israel/313/2008
A/chicken/Saudi Arabia/532/99
A/turkey/Israel/810/2001
A/turkey/Israel/90710/2000
A/chicken/Israel/90658/2000
A/ANHUI/02/2013(H7N9)
A/PIGEON/SHANGHAI/S1069/2013(H7N9)
H7N9
A/falcon/Saudi Arabia/D1795/2005(H5N1)
A/chicken/Darjeeling/170564/2009(H5N1)
A/chicken/India/WB-NIV92456/2009(H5N1)
A/chicken/Bangladesh/1151-10/2010(H5N1)
A/chicken/Bangladesh/1151-11/2010(H5N1)
A/chicken/India/NIV33491/06(H5N1)
A/chicken/Afghanistan/1573-7/2006(H5N1)
A/chicken/Rawalakot/NARC2441A/2006(H5N1)
A/chicken/Afghanistan/1573-65/2006(H5N1)
A/chicken/Sihala/NARC3303.4/2006(H5N1)
H5N1
A/EMPEROR GOOSE/ALASKA/44063-061/2006(H7N9)
A/Chicken/Beijing/1/94
A/Chicken/Hong Kong/G9/97
A/duck/Hong Kong/Y280/97
Y-280 Like
0.01

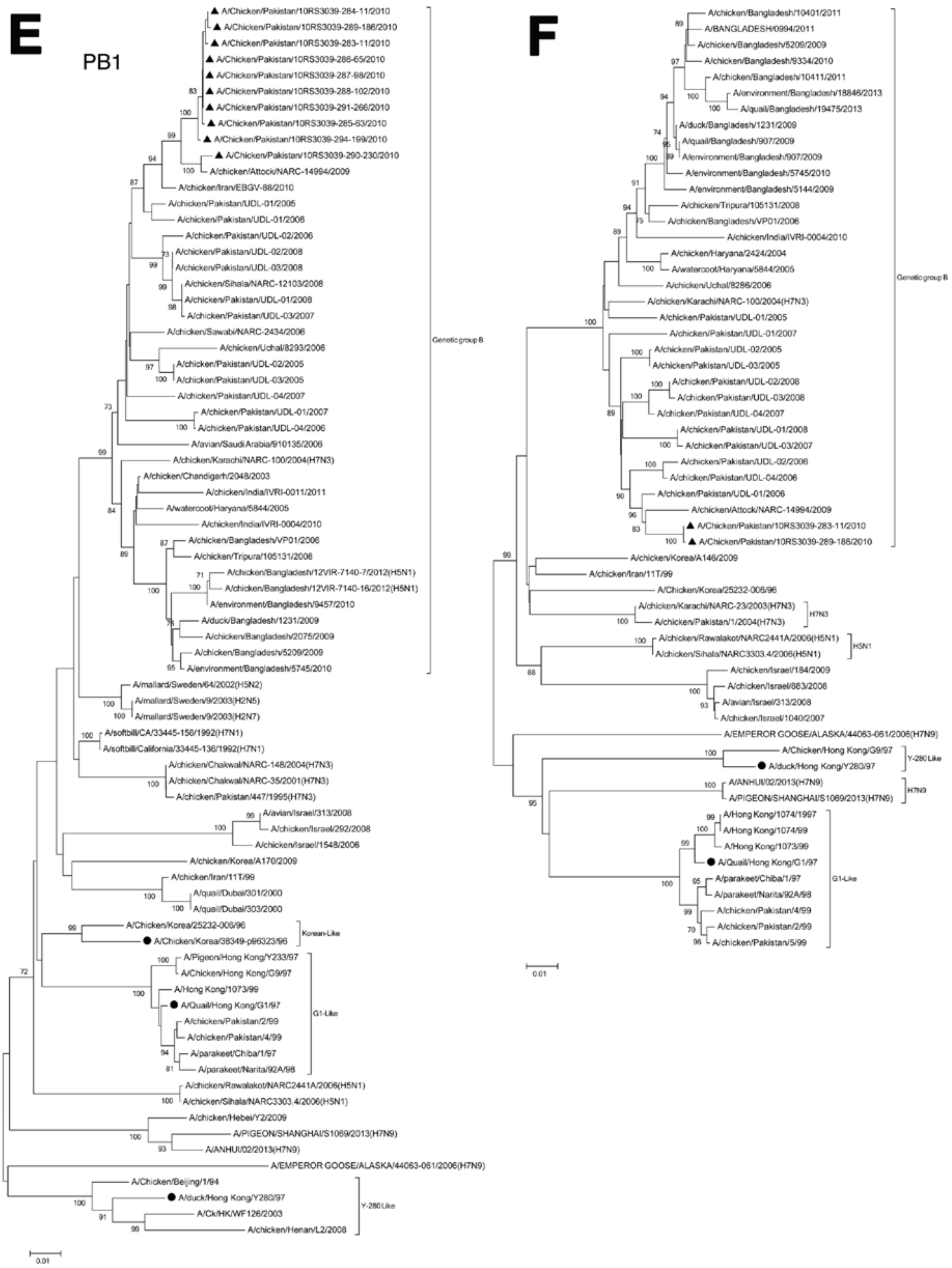
E
PB1
A/Chicken/Pakistan/10RS3039-284-11/2010
A/Chicken/Pakistan/10RS3039-289-186/2010
A/Chicken/Pakistan/10RS3039-283-11/2010
A/Chicken/Pakistan/10RS3039-286-65/2010
A/Chicken/Pakistan/10RS3039-287-98/2010
A/Chicken/Pakistan/10RS3039-288-102/2010
A/Chicken/Pakistan/10RS3039-291-266/2010
A/Chicken/Pakistan/10RS3039-285-63/2010
A/Chicken/Pakistan/10RS3039-294-199/2010
A/Chicken/Pakistan/10RS3039-290-230/2010
A/chicken/Attock/NARC-14994/2009
A/chicken/Iran/EBGV-88/2010
A/chicken/Pakistan/UDL-01/2005
A/chicken/Pakistan/UDL-01/2006
A/chicken/Pakistan/UDL-02/2006
A/chicken/Pakistan/UDL-02/2008
A/chicken/Pakistan/UDL-03/2008
A/chicken/Sihala/NARC-12103/2008
A/chicken/Pakistan/UDL-01/2008
A/chicken/Pakistan/UDL-03/2007
A/chicken/Sawabi/NARC-2434/2006
A/chicken/Uchal/8293/2006
A/chicken/Pakistan/UDL-02/2005
A/chicken/Pakistan/UDL-03/2005
A/chicken/Pakistan/UDL-04/2007
A/chicken/Pakistan/UDL-01/2007
A/chicken/Pakistan/UDL-04/2006
A/avian/Saudi Arabia/910135/2006
A/chicken/Karachi/NARC-100/2004(H7N3)
A/chicken/Chandigarh/2048/2003
A/chicken/India/IVRI-0011/2011
A/watercoot/Haryana/5844/2005
A/chicken/India/IVRI-0004/2010
A/chicken/Bangladesh/VP01/2006
A/chicken/Tripura/105131/2008
A/chicken/Bangladesh/12VIR-7140-7/2012(H5N1)
A/chicken/Bangladesh/12VIR-7140-16/2012(H5N1)
A/environment/Bangladesh/9457/2010
A/duck/Bangladesh/1231/2009
A/chicken/Bangladesh/2075/2009
A/chicken/Bangladesh/5209/2009
A/environment/Bangladesh/5745/2010
Genetic group B
A/mallard/Sweden/64/2002(H5N2)
A/mallard/Sweden/9/2003(H2N5)
A/mallard/Sweden/9/2003(H2N7)
A/softbill/CA/33445-158/1992(H7N1)
A/softbill/California/33445-136/1992(H7N1)
A/chicken/Chakwal/NARC-148/2004(H7N3)
A/chicken/Chakwal/NARC-35/2001(H7N3)
A/chicken/Pakistan/447/1995(H7N3)
A/avian/Israel/313/2008
A/chicken/Israel/292/2008
A/chicken/Israel/1548/2006
A/chicken/Korea/A170/2009
A/chicken/Iran/11T/99
A/quail/Dubai/301/2000
A/quail/Dubai/303/2000
A/Chicken/Korea/25232-006/96
A/Chicken/Korea/38349-p96323/96
Korean-Like
A/Pigeon/Hong Kong/Y233/97
A/Chicken/Hong Kong/G9/97
A/Hong Kong/1073/99
A/Quail/Hong Kong/G1/97
A/chicken/Pakistan/2/99
A/chicken/Pakistan/4/99
A/parakeet/Chiba/1/97
A/parakeet/Narita/92A/98
G1-Like
A/chicken/Rawalakot/NARC2441A/2006(H5N1)
A/chicken/Sihala/NARC3303.4/2006(H5N1)
A/chicken/Hebei/Y2/2009
A/PIGEON/SHANGHAI/S1069/2013(H7N9)
A/ANHUI/02/2013(H7N9)
A/EMPEROR GOOSE/ALASKA/44063-061/2006(H7N9)
A/Chicken/Beijing/1/94
A/duck/Hong Kong/Y280/97
A/Ck/HK/WF126/2003
A/chicken/Henan/L2/2008
Y-280 Like
0.01
F
A/chicken/Bangladesh/10401/2011
A/BANGLADESH/0994/2011
A/chicken/Bangladesh/5209/2009
A/chicken/Bangladesh/9334/2010
A/chicken/Bangladesh/10411/2011
A/environment/Bangladesh/18846/2013
A/quail/Bangladesh/19475/2013
A/duck/Bangladesh/1231/2009
A/quail/Bangladesh/907/2009
A/environment/Bangladesh/907/2009
A/environment/Bangladesh/5745/2010
A/environment/Bangladesh/5144/2009
A/chicken/Tripura/105131/2008
A/chicken/Bangladesh/VP01/2006
A/chicken/India/IVRI-0004/2010
A/chicken/Haryana/2424/2004
A/watercoot/Haryana/5844/2005
A/chicken/Uchal/8286/2006
A/chicken/Karachi/NARC-100/2004(H7N3)
A/chicken/Pakistan/UDL-01/2005
A/chicken/Pakistan/UDL-01/2007
A/chicken/Pakistan/UDL-02/2005
A/chicken/Pakistan/UDL-03/2005
A/chicken/Pakistan/UDL-02/2008
A/chicken/Pakistan/UDL-03/2008
A/chicken/Pakistan/UDL-04/2007
A/chicken/Pakistan/UDL-01/2008
A/chicken/Pakistan/UDL-03/2007
A/chicken/Pakistan/UDL-02/2006
A/chicken/Pakistan/UDL-04/2006
A/chicken/Pakistan/UDL-01/2006
A/chicken/Attock/NARC-14994/2009
A/Chicken/Pakistan/10RS3039-283-11/2010
A/Chicken/Pakistan/10RS3039-289-186/2010
Genetic group B
A/chicken/Korea/A146/2009
A/chicken/Iran/11T/99
A/Chicken/Korea/25232-006/96
A/chicken/Karachi/NARC-23/2003(H7N3)
A/chicken/Pakistan/1/2004(H7N3)
H7N3
A/chicken/Rawalakot/NARC2441A/2006(H5N1)
A/chicken/Sihala/NARC3303.4/2006(H5N1)
H5N1
A/chicken/Israel/184/2009
A/chicken/Israel/883/2008
A/avian/Israel/313/2008
A/chicken/Israel/1040/2007
A/EMPEROR GOOSE/ALASKA/44063-061/2006(H7N9)
A/Chicken/Hong Kong/G9/97
A/duck/Hong Kong/Y280/97
Y-280 Like
A/ANHUI/02/2013(H7N9)
A/PIGEON/SHANGHAI/S1069/2013(H7N9)
H7N9
A/Hong Kong/1074/1997
A/Hong Kong/1074/99
A/Hong Kong/1073/99
A/Quail/Hong Kong/G1/97
A/parakeet/Chiba/1/97
A/parakeet/Narita/92A/98
A/chicken/Pakistan/4/99
A/chicken/Pakistan/2/99
A/chicken/Pakistan/5/99
G1-Like
0.01

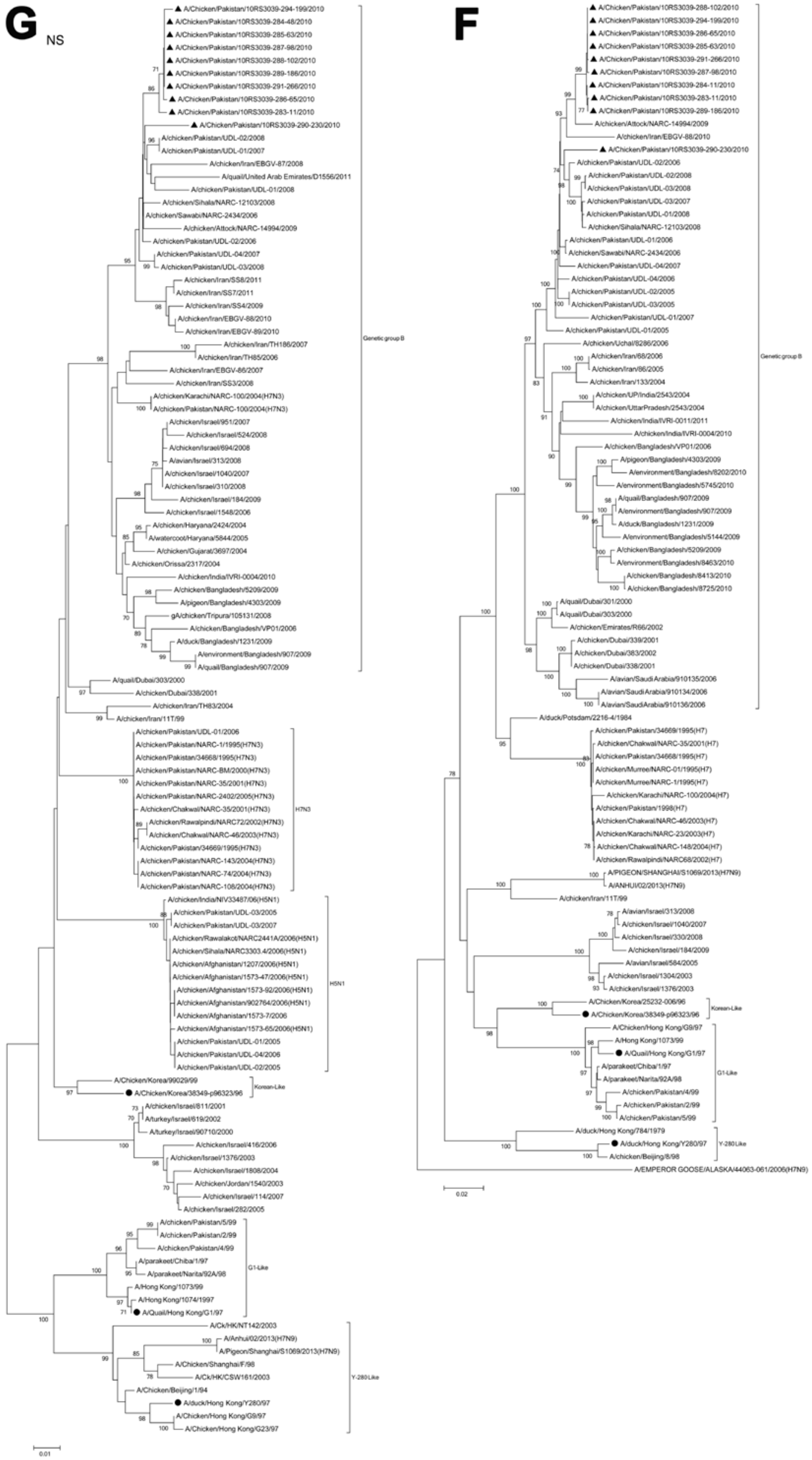
G
NS
A/Chicken/Pakistan/10RS3039-294-199/2010
A/Chicken/Pakistan/10RS3039-284-48/2010
A/Chicken/Pakistan/10RS3039-285-63/2010
A/Chicken/Pakistan/10RS3039-287-98/2010
A/Chicken/Pakistan/10RS3039-288-102/2010
A/Chicken/Pakistan/10RS3039-289-186/2010
A/Chicken/Pakistan/10RS3039-291-266/2010
A/Chicken/Pakistan/10RS3039-286-65/2010
A/Chicken/Pakistan/10RS3039-283-11/2010
A/Chicken/Pakistan/10RS3039-290-230/2010
A/chicken/Pakistan/UDL-02/2008
A/chicken/Pakistan/UDL-01/2007
A/chicken/Iran/EBGV-87/2008
A/quail/United Arab Emirates/D1556/2011
A/chicken/Pakistan/UDL-01/2008
A/chicken/Sihala/NARC-12103/2008
A/chicken/Sawabi/NARC-2434/2006
A/chicken/Attock/NARC-14994/2009
A/chicken/Pakistan/UDL-02/2006
A/chicken/Pakistan/UDL-04/2007
A/chicken/Pakistan/UDL-03/2008
A/chicken/Iran/SS8/2011
A/chicken/Iran/SS7/2011
A/chicken/Iran/SS4/2009
A/chicken/Iran/EBGV-88/2010
A/chicken/Iran/EBGV-89/2010
A/chicken/Iran/TH186/2007
A/chicken/Iran/TH85/2006
A/chicken/Iran/EBGV-86/2007
A/chicken/Iran/SS3/2008
A/chicken/Karachi/NARC-100/2004(H7N3)
A/chicken/Pakistan/NARC-100/2004(H7N3)
A/chicken/Israel/951/2007
A/chicken/Israel/524/2008
A/chicken/Israel/694/2008
A/avian/Israel/313/2008
A/chicken/Israel/1040/2007
A/chicken/Israel/310/2008
A/chicken/Israel/184/2009
A/chicken/Israel/1548/2006
A/chicken/Haryana/2424/2004
A/watercoot/Haryana/5844/2005
A/chicken/Gujarat/3697/2004
A/chicken/Orissa/2317/2004
A/chicken/India/IVRI-0004/2010
A/chicken/Bangladesh/5209/2009
A/pigeon/Bangladesh/4303/2009
gA/chicken/Tripura/105131/2008
A/chicken/Bangladesh/VP01/2006
A/duck/Bangladesh/1231/2009
A/environment/Bangladesh/907/2009
A/quail/Bangladesh/907/2009
Genetic group B
A/quail/Dubai/303/2000
A/chicken/Dubai/338/2001
A/chicken/Iran/TH83/2004
A/chicken/Iran/11T/99
A/chicken/Pakistan/UDL-01/2006
A/chicken/Pakistan/NARC-1/1995(H7N3)
A/chicken/Pakistan/34668/1995(H7N3)
A/chicken/Pakistan/NARC-BM/2000(H7N3)
A/chicken/Pakistan/NARC-35/2001(H7N3)
A/chicken/Pakistan/NARC-2402/2005(H7N3)
A/chicken/Chakwal/NARC-35/2001(H7N3)
A/chicken/Rawalpindi/NARC72/2002(H7N3)
A/chicken/Chakwal/NARC-46/2003(H7N3)
A/chicken/Pakistan/34669/1995(H7N3)
A/chicken/Pakistan/NARC-143/2004(H7N3)
A/chicken/Pakistan/NARC-74/2004(H7N3)
A/chicken/Pakistan/NARC-108/2004(H7N3)
H7N3
A/chicken/India/NIV33487/06(H5N1)
A/chicken/Pakistan/UDL-03/2005
A/chicken/Pakistan/UDL-03/2007
A/chicken/Rawalakot/NARC2441A/2006(H5N1)
A/chicken/Sihala/NARC3303.4/2006(H5N1)
A/chicken/Afghanistan/1207/2006(H5N1)
A/chicken/Afghanistan/1573-47/2006(H5N1)
A/chicken/Afghanistan/1573-92/2006(H5N1)
A/chicken/Afghanistan/902764/2006(H5N1)
A/chicken/Afghanistan/1573-7/2006
A/chicken/Afghanistan/1573-65/2006(H5N1)
A/chicken/Pakistan/UDL-01/2005
A/chicken/Pakistan/UDL-04/2006
A/chicken/Pakistan/UDL-02/2005
H5N1
A/Chicken/Korea/99029/99
A/Chicken/Korea/38349-p96323/96
Korean-Like
A/chicken/Israel/811/2001
A/turkey/Israel/619/2002
A/turkey/Israel/90710/2000
A/chicken/Israel/416/2006
A/chicken/Israel/1376/2003
A/chicken/Israel/1808/2004
A/chicken/Jordan/1540/2003
A/chicken/Israel/114/2007
A/chicken/Israel/282/2005
A/chicken/Pakistan/5/99
A/chicken/Pakistan/2/99
A/chicken/Pakistan/4/99
A/parakeet/Chiba/1/97
A/parakeet/Narita/92A/98
A/Hong Kong/1073/99
A/Hong Kong/1074/1997
A/Quail/Hong Kong/G1/97
G1-Like
A/Ck/HK/NT142/2003
A/Anhui/02/2013(H7N9)
A/Pigeon/Shanghai/S1069/2013(H7N9)
A/Chicken/Shanghai/F/98
A/Ck/HK/CSW161/2003
A/Chicken/Beijing/1/94
A/duck/Hong Kong/Y280/97
A/Chicken/Hong Kong/G9/97
A/Chicken/Hong Kong/G23/97
Y-280 Like
0.01
F
A/Chicken/Pakistan/10RS3039-288-102/2010
A/Chicken/Pakistan/10RS3039-294-199/2010
A/Chicken/Pakistan/10RS3039-286-65/2010
A/Chicken/Pakistan/10RS3039-285-63/2010
A/Chicken/Pakistan/10RS3039-291-266/2010
A/Chicken/Pakistan/10RS3039-287-98/2010
A/Chicken/Pakistan/10RS3039-284-11/2010
A/Chicken/Pakistan/10RS3039-283-11/2010
A/Chicken/Pakistan/10RS3039-289-186/2010
A/chicken/Attock/NARC-14994/2009
A/chicken/Iran/EBGV-88/2010
A/Chicken/Pakistan/10RS3039-290-230/2010
A/chicken/Pakistan/UDL-02/2006
A/chicken/Pakistan/UDL-02/2008
A/chicken/Pakistan/UDL-03/2008
A/chicken/Pakistan/UDL-03/2007
A/chicken/Pakistan/UDL-01/2008
A/chicken/Sihala/NARC-12103/2008
A/chicken/Pakistan/UDL-01/2006
A/chicken/Sawabi/NARC-2434/2006
A/chicken/Pakistan/UDL-04/2007
A/chicken/Pakistan/UDL-04/2006
A/chicken/Pakistan/UDL-02/2005
A/chicken/Pakistan/UDL-03/2005
A/chicken/Pakistan/UDL-01/2007
A/chicken/Pakistan/UDL-01/2005
A/chicken/Uchal/8286/2006
A/chicken/Iran/68/2006
A/chicken/Iran/86/2005
A/chicken/Iran/133/2004
A/chicken/UP/India/2543/2004
A/chicken/UttarPradesh/2543/2004
A/chicken/India/IVRI-0011/2011
A/chicken/India/IVRI-0004/2010
A/chicken/Bangladesh/VP01/2006
A/pigeon/Bangladesh/4303/2009
A/environment/Bangladesh/8202/2010
A/environment/Bangladesh/5745/2010
A/quail/Bangladesh/907/2009
A/environment/Bangladesh/907/2009
A/duck/Bangladesh/1231/2009
A/environment/Bangladesh/5144/2009
A/chicken/Bangladesh/5209/2009
A/environment/Bangladesh/8463/2010
A/chicken/Bangladesh/8413/2010
A/chicken/Bangladesh/8725/2010
A/quail/Dubai/301/2000
A/quail/Dubai/303/2000
A/chicken/Emirates/R66/2002
A/chicken/Dubai/339/2001
A/chicken/Dubai/383/2002
A/chicken/Dubai/338/2001
A/avian/SaudiArabia/910135/2006
A/avian/SaudiArabia/910134/2006
A/avian/SaudiArabia/910136/2006
Genetic group B
A/duck/Potsdam/2216-4/1984
A/chicken/Pakistan/34669/1995(H7)
A/chicken/Chakwal/NARC-35/2001(H7)
A/chicken/Pakistan/34668/1995(H7)
A/chicken/Murree/NARC-01/1995(H7)
A/chicken/Murree/NARC-1/1995(H7)
A/chicken/Karachi/NARC-100/2004(H7)
A/chicken/Pakistan/1998(H7)
A/chicken/Chakwal/NARC-46/2003(H7)
A/chicken/Karachi/NARC-23/2003(H7)
A/chicken/Chakwal/NARC-148/2004(H7)
A/chicken/Rawalpindi/NARC68/2002(H7)
A/PIGEON/SHANGHAI/S1069/2013(H7N9)
A/ANHUI/02/2013(H7N9)
A/chicken/Iran/11T/99
A/avian/Israel/313/2008
A/chicken/Israel/1040/2007
A/chicken/Israel/330/2008
A/chicken/Israel/184/2009
A/avian/Israel/584/2005
A/chicken/Israel/1304/2003
A/chicken/Israel/1376/2003
A/Chicken/Korea/25232-006/96
A/Chicken/Korea/38349-p96323/96
Korean-Like
A/Chicken/Hong Kong/G9/97
A/Hong Kong/1073/99
A/Quail/Hong Kong/G1/97
A/parakeet/Chiba/1/97
A/parakeet/Narita/92A/98
A/chicken/Pakistan/4/99
A/chicken/Pakistan/2/99
A/chicken/Pakistan/5/99
G1-Like
A/duck/Hong Kong/784/1979
A/duck/Hong Kong/Y280/97
A/chicken/Beijing/8/98
Y-280 Like
A/EMPEROR GOOSE/ALASKA/44063-061/2006(H7N9)
0.02

Technical Appendix Figure. Phylogenetic distribution of 10 sequenced viruses (GenBank accession nos. KF975457–KF975503 and KP223678–KP223693) from 280 live poultry retail shops in selected towns of Lahore, Pakistan. The phylogenetic trees were generated by using the neighbor-joining method with maximum composite likelihood using the Tamura-Nei model (*5*) with MEGA (version 5.2.2). Number below branches indicates bootstrap value percentages from 1,000 replicates; bootstrap values of >70% were considered to be significant. Triangle shows viruses collected in current study; circles show the reference lineage viruses. Scale bars indicate nucleotide substitutions per site. Panels shown on subsequent pages: Hemagglutinin (HA); neuraminidase (NA); matrix (M); nucleoprotein (NP); polybasic 1 (PB1) polymerase (PA); nonstructural (NS); polybasic 2 (PB2).

References

1. Cameron KR, Gregory V, Banks J, Brown IH, Alexander DJ, Hay AJ, et al. H9N2 subtype influenza A viruses in poultry in Pakistan are closely related to the H9N2 viruses responsible for human infection in Hong Kong. Virology. 2000;278:36–41. PubMed http://dx.doi.org/10.1006/viro.2000.0585

2. Iqbal M, Yaqub T, Reddy K, McCauley JW. Novel genotypes of H9N2 influenza A viruses isolated from poultry in Pakistan containing NS genes similar to highly pathogenic H7N3 and H5N1 viruses. PLoS ONE. 2009;4:e5788. PubMed http://dx.doi.org/10.1371/journal.pone.0005788

3. Monne I, Ormelli S, Salviato A, De Battisti C, Bettini F, Salomoni A, et al. Development and validation of a one-step real-time PCR assay for simultaneous detection of subtype H5, H7, and H9 avian influenza viruses. J Clin Microbiol. 2008;46:1769–73. PubMed http://dx.doi.org/10.1128/JCM.02204-07

4. Cattoli G, Monne I, Fusaro A, Joannis TM, Lombin LH, Aly MM, et al. Highly pathogenic avian influenza virus subtype H5N1 in Africa: a comprehensive phylogenetic analysis and molecular characterization of isolates. PLoS ONE. 2009;4:e4842 http://dx.doi.org/10.1371/journal.pone.0004842. PubMed

5. Tamura K, Dudley J, Nei M, Kumar S. MEGA4: Molecular Evolutionary Genetics Analysis (MEGA) software version 4.0. Mol Biol Evol. 2007;24:1596–9. PubMed http://dx.doi.org/10.1093/molbev/msm092
